# Environmental variation *and* rivers govern the structure of chimpanzee genetic diversity in a biodiversity hotspot

**DOI:** 10.1186/s12862-014-0274-0

**Published:** 2015-01-21

**Authors:** Matthew W Mitchell, Sabrina Locatelli, Paul R Sesink Clee, Henri A Thomassen, Mary Katherine Gonder

**Affiliations:** Department of Biology, Drexel University, Philadelphia, Pennsylvania 19104 USA; Department of Biological Sciences, University at Albany – State University of New York, Albany, New York 12222 USA; Institut de Recherche pour le Développement (IRD) and Université Montpellier 1 (UM1), 34394 Montpellier, France; Institute of Evolution and Ecology, Eberhard Karls Universität Tübingen, D-72076 Tübingen, Germany

## Abstract

**Background:**

The mechanisms that underlie the diversification of tropical animals remain poorly understood, but new approaches that combine geo-spatial modeling with spatially explicit genetic data are providing fresh insights on this topic. Data about the diversification of tropical mammals remain particularly sparse, and vanishingly few opportunities exist to study endangered large mammals that increasingly exist only in isolated pockets. The chimpanzees of Cameroon represent a unique opportunity to examine the mechanisms that promote genetic differentiation in tropical mammals because the region is home to two chimpanzee subspecies: *Pan troglodytes ellioti* and *P. t. trogolodytes*. Their ranges converge in central Cameroon, which is a geographically, climatically and environmentally complex region that presents an unparalleled opportunity to examine the roles of rivers and/or environmental variation in influencing the evolution of chimpanzee populations.

**Results:**

We analyzed microsatellite genotypes and mtDNA HVRI sequencing data from wild chimpanzees sampled at a fine geographic scale across Cameroon and eastern Nigeria using a spatially explicit approach based upon Generalized Dissimilarity Modeling. Both the Sanaga River and environmental variation were found to contribute to driving separation of the subspecies. The importance of environmental variation differed among subspecies. Gene-environment associations were weak in *P. t. troglodytes,* whereas environmental variation was found to play a much larger role in shaping patterns of genetic differentiation in *P. t. ellioti*.

**Conclusions:**

We found that both the Sanaga River and environmental variation likely play a role in shaping patterns of chimpanzee genetic diversity. Future studies using single nucleotide polymorphism (SNP) data are necessary to further understand how rivers and environmental variation contribute to shaping patterns of genetic variation in chimpanzees.

**Electronic supplementary material:**

The online version of this article (doi:10.1186/s12862-014-0274-0) contains supplementary material, which is available to authorized users.

## Background

**Figure 1:**
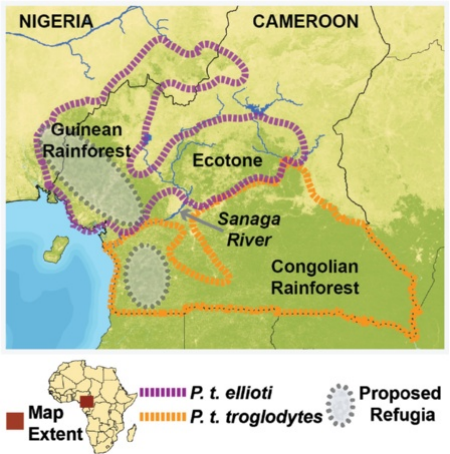
**Overview of the study area.** Important biogeographic features of the lanscape are shown along with the approximate distributions of *P.t. ellioti* (purple) and *P. t. troglodytes* (orange).

The Gulf of Guinea and the Congo River Basin biomes (Figure 1) collectively house ~20% of all plants and animals species, with a very high number of endemic taxa [1]. However, very little is known about the mechanisms that have created this region’s rich biodiversity. Understanding how local factors contributed to generating patterns of genetic differentiation is important to better understand the evolutionary history of tropical taxa. These patterns of genetic differentiation in tropical taxa, and particularly in forest-dwelling primates, may have been shaped by forest history during the Pleistocene, as well as by geographic barriers to dispersal, including rivers and pronounced environmental gradients [2-4].

Tropical Africa is also noted for exhibiting a complex forest history that has undergone considerable change along with the Earth’s climate, and the behavioral ecology of African primates often reflects a shared history with their habitats [5-7]. This relationship has been proposed to have influenced the diversification of rainforest taxa due to the fact that previously continuous populations were isolated from one another in forest “refuges” that persisted during periods of maximum glaciation [5-8]. One of the most widely cited examples to support the ‘Pleistocene Refuge Hypothesis’ are the forests of western Africa, which are divided into two regions located in Upper Guinea and the Gulf of Guinea. Each forest region has its own species assemblages, which are often used to point to the existence of forest refuges in Africa [9]. The Dahomey Gap currently separates these two forest regions and their associated taxa. The Gap is a large, dry, open area that presently extends for about 700 km through Benin, Togo and eastern Ghana [10], but it may have been up to 1,400 km wide during the cool, arid phases of the Pleistocene [3]. Consequently, the Gap has been proposed to have been a barrier to dispersal for several species which resulted in the formation of new species. This process of separation and speciation across the Gap has been proposed to account for the unique faunal assemblages of the Upper and Gulf of Guinea forest blocks, which are both two of the world’s original Global Biodiversity Hotspots [2,4,11,12].

Rivers have also been proposed to play an important role in delimiting the distribution of many species [2,3,10]. For example, the Sanaga River in central Cameroon (Figure 1) has been proposed to delimit the distributions of several pairs of primate species and subspecies: *Mandrillus leucophaeus*/*M. sphinx*, *Cercopithecus erythrotis*/*C. cephus*, *C. nictitans martini*/*C. n. nictitans*, *C. pogonias pogonias*/*C. p. grayi*, *P. t. ellioti*/*P. t. troglodytes* and possibly, *Gorilla gorilla diehli*/*G. g. gorilla* [1,13-18]. The Congo separates chimpanzees (*Pan troglodytes*) from bonobos (*P. paniscus*) [15], and the Ubangi, Niger and Sanaga Rivers in central Africa may be important in delimiting chimpanzee subspecies from one another. However, the role of riverine barriers in shaping patterns of diversity across the landscape are poorly understood [4,19]. Changes in river size and course over time may dramatically affect the significance of a given river to act as a barrier to dispersal, and habitat changes in the vicinities of rivers may confound distiguishing between the role of the river and changes in the environment [4]. The Sanaga River, for instance, has also been proposed to act as an historical boundary that separates *P. t. ellioti* and *P. t. troglodytes* [20-23], but its significance is questionable [23]. Little is known about the history of the Sanaga, but it is entirely possible that its course and size have changed over time, especially given this region’s sensitivity to climatic oscillations [9,24]. And although chimpanzee genetic populations are seemingly partitioned along the location of the Sanaga, there is evidence of gene flow occurring between these populations [22,23,25].

Until very recently, the role of ecological gradients in driving the diversification of tropical taxa has been under-appreciated. Ecological gradients are zones of transition between habitat types that display marked differences in ecological variables (i.e. precipitation, temperature, vegetation density, etc.) across their range. These gradients have been linked with driving adaptive variation in several taxa in different parts of the world, for example, suture zones in Australia [26] and more recently in Cameroon [27]. In this ecological gradient model, speciation across habitat gradients is driven by local adaptation, whereas the genetic differentiation of allopatric populations is driven by genetic drift resulting from historical isolation in refugia or separation by geographic barriers, including rivers and other features of the landscape, while the habitats remain generally the same [28]. A prominent gradient is present in central Cameroon, which transitions from Guinean forest in the west to Congolian rainforest in the south and to Sahelian habitats in the north and east [29] (Figure 1). This forest-savanna mosaic was termed by Smith *et al.* [30] as an ‘ecotone’. A growing body of evidence suggests that this ecotone has been important in promoting the evolutionary diversification of insects [31], reptiles [27] and birds [30,32]. Complementary genetic datasets for mammals occupying this region remain sparse, which makes it difficult to distinguish between the relative influence of environmental and topographic factors in governing diversification and population structuring of mammals, particularly primates where distributional data are generally the only information available.

Recent analyses suggest that the two subspecies of chimpanzees present in Cameroon may be divided into genetically distinct populations: *P. t. troglodytes* occurs south of the Sanaga River, while *P. t. ellioti* occurs north. *P. t. ellioti* may be further subdivided into two additional populations: a population that occurs in forested regions of western Cameroon, *P. t. ellioti* (Rainforest), and a second population that occupies central Cameroon, *P. t. ellioti* (Ecotone) [23]. While it appears that the Sanaga River has played an important role in separating these subspecies, the estimated ranges of these populations coincide with the transition of the two rain forests types and the ecotone [23], and all three populations occupy significantly different habitat types [33]. This suggests that a relationship exists between the environmental variation and the partitioning of genetic variation in chimpanzees found across the study area. Taken together, these factors make it difficult to distinguish between the relative importance of the Sanaga River or habitat variation in shaping the partitioning of chimpanzee population genetic variation given their close proximity to one another (Figure 1).

The available genetic data for wild chimpanzees sampled across this region consist of 21 autosomal microsatellite loci and the HVRI region of the mtDNA [23]. Since these loci meet expectations of neutrality, the genetic data set cannot be used to directly examine the role of adaptation in shaping patterns of variation in chimpanzees across the study area [27,34]. However, it is still possible to use these neutrally-evolving genetic makers to infer whether environmental factors other than, or in addition to, the Sanaga have contributed to shaping the patterning of genetic variation found in chimpanzees from the region, indicating that chimpanzees in Cameroon and Nigeria may follow a pattern of isolation-by-environment, a relationship driven by selective evolutionary processes [35].

Table 1 lists expected patterns of genetic variation depending upon whether isolation across the Sanaga River or environmental variation occupies a dominant role in shaping patterns of genetic variation in chimpanzees across the study region. Collectively, these predictions are related to: (*i*) the diversity and distribution of alleles; (*ii*) the dates of divergence between populations; (*iii*) the location of barriers to gene flow; and (*iv*) the demographic characteristics of populations (e.g. population size and population growth). This study is an extension of a complimentary paper [23] which looked at the population genetic structure and demographic history (*ii* and *iv*) of chimpanzees in Cameroon and Nigeria. That study found evidence that positive selection likely plays a role in shaping patterns of chimpanzee genetic diversity, and that this may be the results of landscape variation. This study is the next step, and focuses on using a spatially explicit modeling technique, Generalized Dissimilarity Modeling (GDM) [36], to quantify and visualize associations between landscape variation and genetic variation in chimpanzees. The results of the analyses presented here were used to support or reject predictions regarding spatial patterns of genetic diversity (*i*) and the location of barriers to gene flow (*iii*).

**Table 1:** **Models to explain the partitioning of chimpanzee genetic variability across the study region, and associated predictions**

**Predictions**	**Riverine barriers**	**Ecological variation**
**Diversity of neutral alleles**	Highest on opposite banks of Sanaga and Mbam Rivers	Significant correlations between the distribution of allelic diversity and variation in one or more environmental variables.
**Population divergence (T**_**MRCA**_ **)**	Broad spectrum of time intervals	Broad spectrum of time intervals
**Barriers to gene flow are located**	At the Sanaga, with highest resistance at the Sanaga Delta and decreasing towards headwaters	At or near ecotone boundaries, but not at adjacent Guinean-Congolian rainforest boundary in western Cameroon
**Population history includes**	Demographic stability, other scenarios possible	Demographic stability, other scenarios possible

## Results and discussion

### Regional patterns of isolation-by-environment

**Figure 2:**
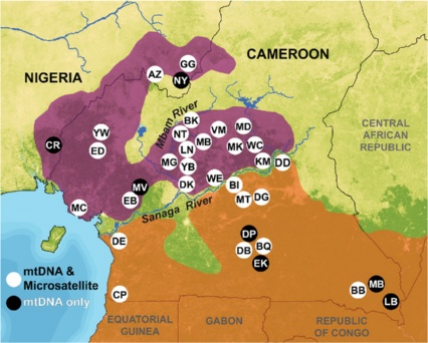
**Sample locations of chimpanzees included in the study.** Locations spanned Cameroon and eastern Nigeria. Approximate distributions of *P. t. ellioti* (purple) and *P. t. troglodytes* (orange) ranges are shown. White circles denote both mtDNA and microsatellite data were available at the location. Black circles denote only mtDNA data was available.

The data set used for analysis was used in a previous study on the population genetic structure of chimpanzees [23]. The genetic data consisted of autosomal microsatellite genotype profiles of 187 unrelated individuals sampled from 28 locations across eastern Nigeria and Cameroon and 604 mtDNA sequences sampled from 35 locations across the study region (Figure 2). These data were subjected to rigorous quality control procedures when generated, including separate DNA extractions and several independent calculations of allele sizes [23]. All loci met expectations of neutral evolution, as measured by the results of an outlier test [37] and meeting expectations of Hardy-Weinberg equilibrium [23]. We ran GDM’s using microsatellite genotypes and mtDNA sequencing data in order to assess the relative contribution of spatial variables to patterns of genetic differentiation. This method fits genetic distance matrices from both microsatellite (*F*_*ST*_) and mtDNA data against straight-line geographic distance, topographic, climatic and vegetation variables assumed to contribute to chimpanzee habitat ecology and biogeographic boundaries. We ran models using: (*i*) geographic distance between sample locations only; (*ii*) environmental variables only (topography, climate and vegetation); (*iii*) environmental variables and geographic distance between sample locations, and (*iv*) environmental variables, distance and riverine barrier layers (‘complete barrier’ layers and resistance surfaces). The percent of genetic variation accounted for by the microsatellite genotype data and the mtDNA data for three different classes of predictor variables are shown in Table 2.

**Table 2:** **Region wide gene-environment relations**

**Genetic dataset**	**GDM model**			
	**Distance only** ^**a**^	**Environment only** ^**b**^	**Environment + Distance** ^**c**^	**Environment + Distance + Rivers**
Autosomal Microsatellites (*F*_ST_)	4	10	11	13
mtDNA – Pairwise Differences	6	22	-	56
mtDNA – Tamura and Nei	11	32	-	72

^a^Model includes only geographic distance between sample locations as a predictor variable.

^b^Model includes environmental data layers as well as resistance matrices, which incorporate river barriers and habitat suitability, as predictor variables. For all models, resistance matrices were not included, as they had no significant contribution.

^c^Entries are blank because geographic distance was not a significant contributor to the final model.

**Figure 3:**
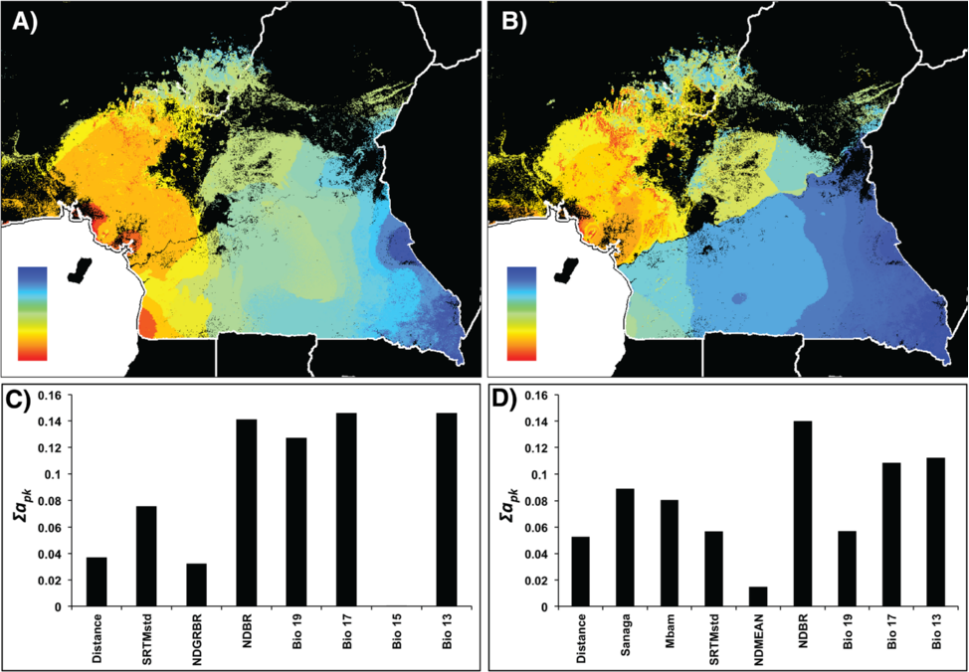
**Spatial predictions of, and contributing variables to, microsatellite differentiation using GDM.** Spatial predictions of genetic differentiation based on microsatellite diversity (*F*_*ST*_) using environmental variables and distance **(A and C)** and environmental variables, distance and rivers **(B and D)**. Colors between maps are not comparable. Within maps, areas with similar colors along color gradients are predicted to be more similar genetically. Panels **C** and **D** represent the relative importance of the selected variables that significantly contribute to the models. Each panel explains the map directly above it.

Pairwise differences (*F*_*ST*_) between sample locations at autosomal microsatellites are shown in Additional file 1. The correlation between *F*_*ST*_ and predictor variables was then interpolated across the study area using the GDM method, which revealed that geographic distance alone accounted for 4% and environmental variation alone accounted for 10% of the observed genetic variation (Table 2). A combination of geographic distance and environmental variation only increased the explanatory power of the model to 11% (Table 2), even though distance was a significant contributor to the model. These findings are consistent with the results of from a previous study [23], that revealed that genetic population structure in chimpanzees in the region can only be weakly explained by a pattern of isolation-by-distance. In the model including geographic distance and environmental variables jointly, genetic differentiation was predicted along a general west–east cline from Guinean rainforest to ecotone to Congolian rainforest (Figure 3a). This model was mostly explained by minimum NDVI during the yearly period with the least amount of new vegetation (NDBR) and climatic variables relating to precipitation, and to a lesser extent by slope and geographic distance (Figure 3c).

When we included the Sanaga and Mbam River layers, both rivers significantly contributed to the partitioning of genetic variation but the total variation explained increased to only 13% in that particular model (Table 2). The cost-resistance surface layer of riverine disperal barriers did not significantly contribute to the population structure of chimpanzees across the study region. The pattern of predicted genetic differentiation changed and featured a sharp divide at the Sanaga, a less pronounced, but still obvious divide across the Mbam, and a distinct gradient across elevations in the Cameroon Highlands (Figure 3b). The relative contribution of the environment variables was similar to the previous model, with the Sanaga and Mbam rivers contributing only moderately (Figure 3d).

**Figure 4:**
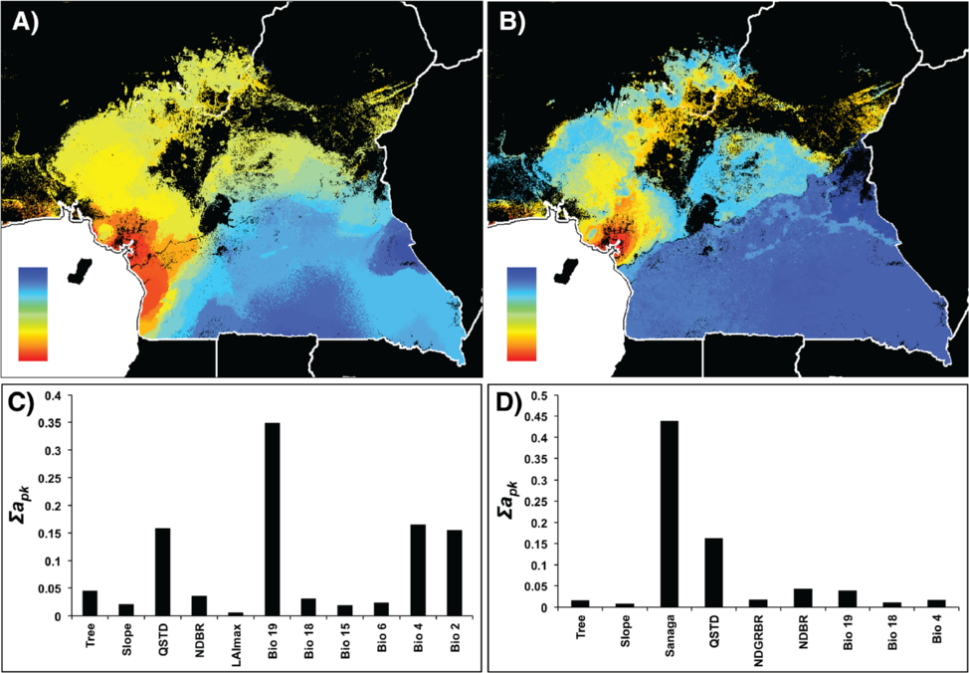
**Spatial predictions of, and contributing variables to, mtDNA differentiation using GDM.** Spatial predictions of genetic differentiation based on mtDNA diversity (pairwise differences) using environmental variables and distance **(A and C)** and environmental variables, distance and rivers **(B and D)**. Colors between maps are not comparable. Within maps, areas with similar colors along color gradients are predicted to be more similar genetically. Panels **C** and **D** represent the relative importance of the selected variables that significantly contribute to the models. Each panel explains the map directly above it.

Pairwise differences between sample locations at the mtDNA HVRI locus are shown in Additional file 2 and Tamura and Nei indices [38] are shown in Additional file 3. The response between pairwise distance and the predictor variables was interpolated across the study area using the GDM method, which revealed that geographic distance alone accounted for 6% and environmental variation alone accounted for 22% of the observed genetic variation (Table 2). The response between Tamura and Nei distance and the predictor variables was also calculated, which revealed that geographic distance alone accounted for 11% and environmental variation alone accounted for 32% of the observed genetic variation (Table 2). Geographic distance did not significantly contribute to the partitioning of genetic variation when either model was run combined with environmental variation. When this analysis was run with only environmental variables, genetic differentiation was predicted along two pronounced clines, one from coastal rainforest to montane forests and ecotone, and another from coastal rainforest and ecotone southward to Congolian rainforest (Figure 4a).

When the Sanaga and Mbam River layers were included, the amount of genetic variation accounted for by the pairwise distance model increased to 56%, while the Tamura and Nei model increased to 72%, the highest percentage for all models across the entire study area included in this study (Table 2). Again, as with the microsatellite data, the cost-resistance surface layer of riverine disperal barriers did not significantly contribute to the model. Genetic differentiation was predicted across a sharp divide at the Sanaga River, with further differentiation occurring along elevational gradients in western Cameroon and eastern Nigeria (Figure 4b). The Sanaga River was by far the most important contributor, with variability in several environmental factors (e.g. surface moisture, temperature, precipitation and diurnal range) moderately contributing to the overall partitioning of genetic diversity (Figure 4d).

The Sanaga River is the most important contributor to the partitioning of mtDNA genetic diversity, and the model using both river layers accounted for the greatest amount of the genetic variation of all the region-wide models (Table 2, Figure 4). This result is unsurprising, especially given the results of a previous study, showing a deep break of mtDNA haplotypes across the Sanaga [23]. The GDMs for microsatellites suggest that rivers play an important, but incomplete, role in separating *P. t. ellioti* and *P. t. troglodytes* from one another. While the Sanaga and Mbam Rivers contributed significantly to the analysis from microsatellite genotypes, these rivers were never the top contributors, and they only slightly increased the overall explanatory power of the models. These GDMs showed the strongest associations with different habitat types (Figure 3). These results, taken together, show that the Sanaga River is indeed a contributor to the partitioning of genetic diversity of chimpanzees in the region. However, given the importance of environmental variables in the microsatellite models, it is apparent that environmental variation also contributes to shaping patterns of genetic diversity in chimpanzees in the region.

### Intra-population patterns of isolation-by-environment

**Figure 5:**
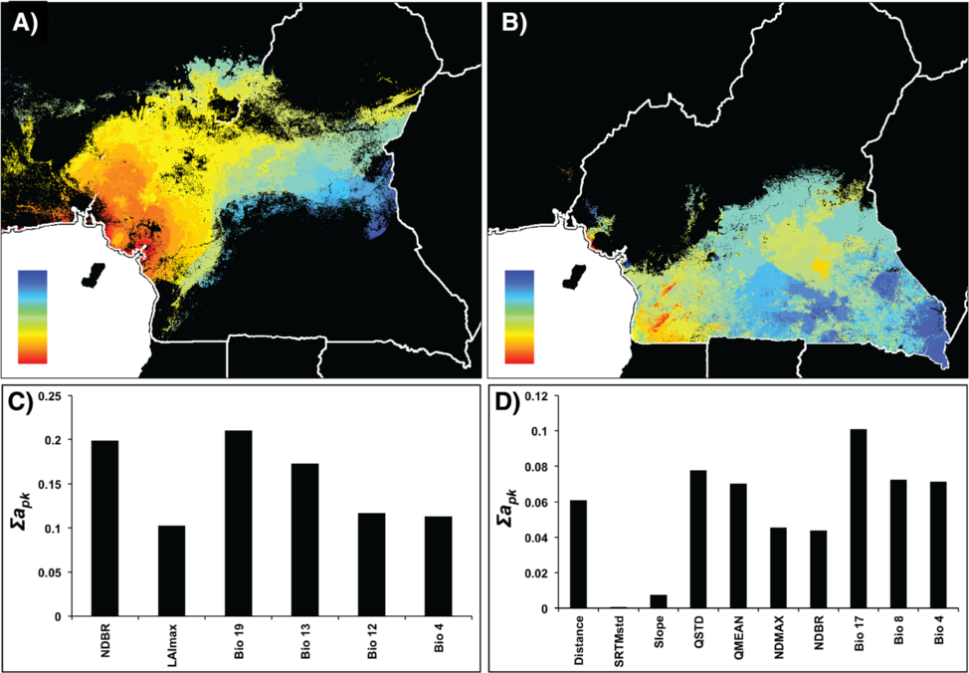
**Intra-population spatial predictions of, and contributing variables to, microsatellite differentiation using GDM for two populations.** Spatial predictions of genetic differentiation based on microsatellite diversity (*F*_*ST*_) using environmental variables, distance, and river layers for *P. t. ellioti* **(A and C)** and *P. t. troglodytes* **(B and D)**. Colors between maps are not comparable. Within maps, areas with similar colors along color gradients are predicted to be more similar genetically. Panels **C** and **D** represent the relative importance of the selected variables that significantly contribute to the models. Each panel explains the map directly above it.

We completed additional GDMs to assess the partitioning of genetic variation among populations within subspecies, by including a two-population grouping and a three-population grouping [33] based on cluster analysis from a previous study [23]. We ran these models only using microsatellite genetic differentiation as estimated by *F*_*ST*_. We completed the analysis for the *P. t. ellioti* group using environmental variables, geographic distance, and the Mbam River layer. This model accounted for 20% of the observed genetic variation (Table 3). Neither geographic distance nor the Mbam River significantly contributed to this model, although a model run with geographic distance alone could explain 6% of genetic variation (Table 3). Genetic differentiation was predicted along a gradual west–east cline from the Guinean Rainforest to the ecotone of central Cameroon (Figure 5a). A variety of environmental variables were important contributors, including NDVI of the least green season, precipitation variables, temperature seasonality and leaf area index (Figure 5c).

**Table 3:** **Percent of genetic variation within chimpanzee populations explained by GDM** ^**a**^

**Chimpanzee Population** ^**b**^	**Two-population model**		
	**Distance only** ^**c**^	**Environment only** ^**d**^	**Environment + Distance** ^**e**^
*P. t. ellioti* ^f^	6	20	-
*P. t. troglodytes*	12	42	48
	**Three-population model**		
	**Distance only**	**Environment only**	**Environment + Distance**
*P. t. ellioti* (Rainforest)	2	91	-
*P. t. ellioti* (Ecotone)	1	37	-
*P. t. troglodytes*	12	42	48

^a^In order to examine genetic diversity within populations, only microsatellite data (*F*_*ST*_) was used.

^b^Sub-groupings of chimpanzee populations correspond to distinct genetic populations as determined by Mitchell *et al.* [23]. The *P. t. troglodytes* group was included in both the two- and three-population model, as only the *P. t. ellioti* group is sub-divided in the three-population model.

^c^Model includes only geographic distance between sample locations as a predictor variable.

^d^Model only includes environmental data layers as predictor variables.

^e^Entries are blank because geographic distance was not a significant contributor to the final model.

^f^A least cost path layer for the Mbam River was included when running the *P. t. ellioti* two-population model, but as it was not a significant contributor, was not included in this table.

We completed the analysis for the *P. t. troglodytes* group using environmental variables and geographic distance as response variables. Geographic distance alone accounted for 12% and environmental variation accounted for 42% of observed genetic variation. Combining geographic distance and environmental variation as predictors increased the explanatory power of the model to 48% (Table 3). Geographic distance was a significant contributor to the overall partitioning of genetic variation found in *P. t. troglodytes*, although a pocket of differentiation occurred in southwest Cameroon that did not appear to follow the clinal model found across the rest of this subpsecies’ range in southern Cameroon (Figure 5b). In addition to geographic distance, several environmental variables relating to precipitation, temperature and surface moisture (annual mean and variability), were also associated with genetic variation in *P. t. troglodytes* across the study area (Figure 5d).

**Figure 6:**
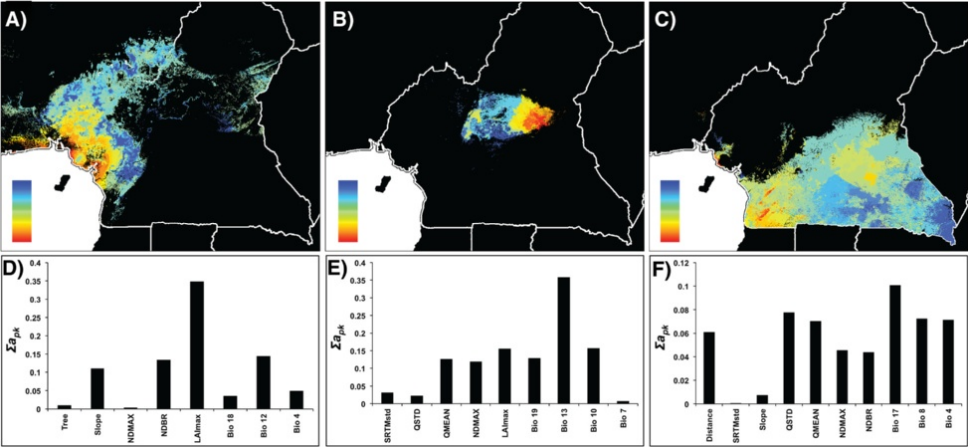
**Intra-population spatial predictions of, and contributing variables to, microsatellite differentiation using GDM for three populations.** Spatial predictions of genetic differentiation based on microsatellite diversity (*F*_*ST*_) using environmental variables and distance for *P. t. ellioti* (Rainforest) **(A and D)**, *P. t. ellioti* (Ecotone) **(B and E)** and *P. t. troglodytes* **(C and F)**. Colors between maps are not comparable. Within maps, areas with similar colors along color gradients are predicted to be more similar genetically. Panels **D-F** represent the relative importance of the selected variables that significantly contribute to the models. Each panel explains the map directly above it.

Environmental variation is strongly correlated with the partitioning of genetic diversity in *P. t. ellioti*. The analysis of the rainforest population of *P. t. ellioti* revealed that environmental variables accounted for 91% of the observed genetic variation (Table 3), particularly differences in vegetation, precipitation and slope (Figure 6d). Geographic distance was not a significant contributor to the model when combined with the environmental variables, and could only account for 2% of the genetic variation when included as the sole predictor. Genetic differentiation was predicted along a gradient that spans coastal to montane forests (Figure 6a), consistent with other analyses that included all of *P. t. ellioti*.

Environmental variables accounted for 37% of the partitioning of genetic variation of the *P. t. ellioti* (Ecotone) population (Table 3). Geographic distance was not a significant contributor when combined with the environmental variables and could only account for 1% of the genetic variation when included as the sole predictor. In addition, genetic differentiation followed a west–east gradient, across a wide breadth of habitats that includes a forest-savanna mosaic that spans central Cameroon (Figure 6b). The most important contributors to influencing the partitioning of genetic variation among chimpanzees located in central Cameroon include precipitation variables, surface moisture and vegetation density (Figure 6e), which are all important variables that define forest and savanna habitats.

Significant environmentally associated gradients were observed within all tested populations north of the Sanaga River (*P. t. ellioti* and its two sub-groupings). In western Cameroon and eastern Nigeria elevational gradients were once again observed to account for the highest proportion of variation (Figures 5a, and c, 6a and d). In central Cameroon, genetic differentiation was predicted across habitats with variability in vegetation density, moisture and precipitation (Figure 6b and 6e). In addition, *P. t. ellioti* habitats in the forested regions of western Cameroon are characterized by steep, densely forested areas with pronounced precipitation seasonality in northwest Cameroon, and a wider breadth of habitats that include both forest and savanna that experience even more seasonal variability in temperature and precipitation [32,33]. In southern Cameroon, geographic distance was a major predictor, and there was no apparent spatial association between genetic diversity and environmental clines (Figure 5b and 6e). These patterns are unsurprising, especially given that ecological niche models show that *P. t. troglodytes* habitat are relatively homogeneous in terms of elevation, temperature and precipitation [33]. In contrast, habitat variability increases dramatically north of the Sanaga River [27,29,32,33,39].

### Hypothesis testing

Using the geospatial modeling approaches presented here, we can provide preliminary assessments of the likely contributors that underlie the partitioning of chimpanzee population genetic diversity: Riverine barriers and environmental variation. Overall, predicted patterns of neutral allele diversity across the study region were found to be relatively consistent with each other in both of the region-wide models (Figures 3 and 4). Without the river layers, the models both showed a pronounced association between allele diversity and habitat type, with a general west–east gradient that might be consistent with differentiation between Guinean Rainforest – ecotone – Congolian Rainforest habitat types.

How do rivers contribute to generating the pattern found in chimpanzees across the study area? Results from previous work show a distinct division of population clusters precisely at the location of the Sanaga River. However, the Sanaga is an incomplete boundary, as there is evidence of migration across the river [12,21-23,25]. Population genetic analysis shows that chimpanzees across the Sanaga River exchange migrants at a rate of approximately 1 migrant per generation, lending even more support to the face that the Sanaga River is not a complete boundary to gene flow [23]. Including the river layers increased the explanatory power of both region-wide models presented in this study (Table 2). The Sanaga River is always an important contributor to patterns of region-wide genetic differentiation, but never the only one (Figures 3 and 4), and in some cases not even the most important (Figure 3). Additionally, the Mbam River was not found to be a significant contributor to genetic differentiation north of the Sanaga River (Figure 6a and c).

How does environmental variation contribute to chimpanzee genetic diversity? Although variation in forest cover, preciptation, and slope are generally more important across the study area than previously appreciated, these variables impact *P. t. troglodytes* and *P. t. ellioti* differently. Amongst *P. t. troglodytes* south of the Sanaga, the distribution of allelic diversity does not appear to be associated with any environmental clines, and instead, appears to follow a pattern of isolation-by-distance (Figures 3 and 4). In contrast, the partitioning of genetic variation in *P. t. ellioti* appears to be substantially influenced by changes in the environment (Figures 3 and 4). Finally, these associations between habitat and the partitioning of genetic variation are especially noticeable when the data for *P. t. elloti* are split into two subpopulations in the western rainforest and central ecotone regions (Figures 5 and 6), in which environmental varation accounts for 91% and 37%, respectively, of the variation explained by the GDM for this subspecies. These observations provide support for the hypothesis that habitats play an important role in structuring chimpanzee populations.

The analyses presented here show, for the first time, that an important relationship exists between the partitioning of genetic variation in chimpanzees and environmental variation in Cameroon and eastern Nigeria, particularly regarding changes in slope, climate and vegetation. Characterizing these relationships is important because it might explain why the separation of chimpanzee subspecies across the Sanaga only partially explains the distribution of genetic variation and that the role of adaptation to local environmental conditions may be substantially underappreciated in the evolution of chimpanzee subspecies. Finally, these findings suggest that the role of environmental variation may be under-appreciated in other primates whose distributions may have been influenced by the Sanaga River [13,14]. Future studies that examine patterns of isolation-by-environment [35] in primates and other mammals that occur sympatrically with chimpanzees may reveal that the differentiation across the ecotone is a more common feature of these species’ evolutionary history than previously believed.

## Conclusions

Although this study focused on quantifying a pattern of isolation-by-environment in chimpanzees using genetic data comprised of a relatively small number of neutral loci, several important conclusions have emerged from this analysis. The Sanaga River is an important contributor to patterns of genetic diversity in chimpanzees in Cameroon (Figure 4). However, it is not the only contributing factor (Figures 3 and 4). Habitat and elevational gradients play a major role in partitioning genetic differentiation, especially in *P. t. ellioti*. This was especially evident when GDMs were run for sub-groupings of chimpanzee populations (Figures 5 and 6), where both forest type across elevational and precipitation/moisture gradients played a major role, which is consistent with the ecological niches inhabited by each sub-population.

Future studies that include data from loci that might be subject to selection are needed to better understand these complex associations, as this study is limited in its ability to detect environmentally diven natural selection in chimpanzees. Genome-wide single nucleotide polymorpism (SNP) data, for example, will allow for quantifying these associations, and provide improved spatial and temporal resolution to disentangle the relative role of Pleistocene refugia in generating genetic diversity. The evolutionary impacts of the environmental gradient in Cameroon have been examined in a small number of taxa [27,31,32], but for the most part, there are not enough studies examining the relative roles of ecotones versus other biogeographic barriers (i.e. the Sanaga River).

In addition to being an important region for chimpanzees, there are also a number of other primate species and subspecies that are presumably influenced by the Sanaga River, including: *Mandrillus leucophaeus*/*M. sphinx*, *Cercopithecus erythrotis*/*C. cephus*, *C. nictitans martini*/*C. n. nictitans*, and *C. pogonias pogonias*/*C. p. grayi* [1,13-15,21]. Because these pairs of primate taxa occupy vastly different niches [13,14], it is important to also investigate the role that environmental variation might play in shaping their own patterns of genetic diversity. This study provides an important first step in this process by showing that chimpanzee population structure in eastern Nigeria and Cameroon is not solely driven by separation across riverine barriers, as previously thought.

## Methods

### Overview of modeling patterns of isolation-by-environment

There are many spatially explicit genetic analysis methods that can interpolate a population’s genetic structure across a study region [34,40-45]. Recent advances in these methods and the increasing number of publicly-available remote sensing data sets have improved evaluating how ecological, geological and environmental variables influence the genetic structure of populations [34]. Not only can these new models quantify statistical associations of genetic differentiation of sampled populations of study taxa and their habitats, but they can also be used to project inferred patterns of diversity across unsampled areas of the study taxa’s projected habitat [34]. These continuous projections represent expected genetic or phenotypic variation, given environmental and/or topographic variation [34]. These models have been used to answer questions from a wide range of topics, including conservation prioritization [46,47], disease ecology [48-51], and biological diversification and speciation [27,32,52,53].

There are a variety of spatial modeling techniques, which include simple regression methods, such as spatially auto-correlated principal components analysis [54], and random forests [55,56] that are used to evaluate environmental and biodiversity associations. GDM is a versatile technique that was originally developed as a matrix regression technique used to study species beta diversity [36]. GDM has also been used to study the relationship between environmental matrices and matrices of morphological and genetic turnover [27,32,34]. In brief, GDM evaluates dissimilarities between environmental and topographic ‘predictor’ variables and ‘response’ variables, which can include pairwise genetic distances (e.g. *F*_*ST*_) or morphological diversity among populations. The relationships between these types of variables are often non-linear, such as pairwise genetic distances, which are scaled between 0–1, while environmental variables may increase or decrease beyond this scale. GDM’s use of non-linear regression algorithms is especially appropriate for understanding the complex influences that landscape features have on shaping patterns of genetic diversity [34].

### Data curation and generation

We used DNA from non-invasively collected chimpanzee hair and fecal samples from a previous study [23]. All samples from this study were transported from Cameroon to the United States in full compliance with Convention of International Trade in Endangered Species of Wild Fauna and Flora (CITES) and Center for Disease Control (CDC) export and import regulations. This research was carried out with IACUC approval from the University at Albany – State University of New York. The genetic data included in the analysis consisted of 21 autosomal microsatellite loci, all determined to be selectively neutral, as well as a 501-base pair (bp) fragment spanning the hypervariable D-loop of mitochondrial (mt) DNA [23]. One hundred and eighty seven individuals from 28 sampling locations found across the study region of Cameroon and eastern Nigeria were genotyped at 21 microsatellite loci (Figure 2). We also included 354 mtDNA control region sequences from 35 sampling locations found across the study region (Figure 2).

For the microsatellite data set, we calculated pairwise *F*_*ST*_ values between sample locations using Arlequin version 3.5 [57]. Each pairwise genetic distance was determined by 100,000 replications. In addition, we calculated pairwise difference and Tamura and Nei [38] indices using mtDNA sequence data, from previous studies [20,22,23,58,59], between sample locations from eastern Nigeria and Cameroon using Arlequin version 3.5 [57]. We used a 5:1 transition to transversion weight [22,60], and each estimate was determined by 100,000 replications. All microsatellite loci and mtDNA haplotypes met expectations of Hardy-Weinberg equilibrium and were found to be selectively neutral by an outlier analysis [23].

We obtained environmental data layers from several remote sensing platforms to characterize the habitats available to chimpanzees across the study region. These layers are described in Additional file 4 and can be grouped into three broad categories of factors that describe variation in topography, climate, and vegetation. The topography of the region was described by several variables, including elevation and slope, each sampled at 1 km resolution from the Shuttle Radar Topography Mission dataset [33,61]. We also mapped and included major rivers across the study area in a hydrography layer [62]. Climatic factors describe annual variation in seasonality, temperature and precipitation. Eighteen layers were obtained from WorldClim 1.4 [63] and sampled at 1 km resolution. The category of variables shown in Additional file 4 describes a series of vegetation factors. These factors describe variation in ground cover across the study region. We calculated percent Tree Cover and Leaf Area Index, two measures of vegetation density, from Moderate Resolution Imaging Spectroradiometer (MODIS) imagery [64,65]. Two layers included in this category describe surface moisture, leaf water content and deciduousness of vegetation (QMEAN and QSTD) as measured from the Quick Scatterometer satellite data set [66]. These layers represent annual mean and variability (standard deviation) of surface moisture. Finally, we described variation in vegetation density and deciduousness of vegetation across the study area using a series of Normalized Difference Vegetation Indices, or NDVI, layers [27]. NDVI measurements include mean annual NDVI (NDMEAN), maximum annual NDVI (NDMAX), maximum NDVI during the yearly period of the commencement of the rainy season when new vegetation occurs (NDGR), minimum NDVI during the yearly period with the least amount of new vegetation (NDBR), and NDVI seasonality (NDGRBR).

In order to run the model as accurately as possible, we restricted all spatial analysis to areas within Cameroon and eastern Nigeria where chimpanzees are likely to occur at present time. We used an ecological niche model (ENM) of chimpanzees specifically created for chimpanzees in the study region [33] that was created using a maximum entropy method applied in the program MAXENT [67] to approximate chimpanzee occurrence. This particular model is the most extensive to date for chimpanzees in our region of study, drawing upon occurrence points from the ranges of *P. t. ellioti* (656 total) and *P. t. troglodytes* (98 total). We used a logistic distribution of values from the ENM to establish a threshold in which all cells with a value of less than 10% suitability were excluded from the training mask used as a background for all GDMs, based on a visual comparison of the maps. We excluded these cells to accurately estimate the known range of chimpanzees in the region [68]. By excluding all cells with a value of less than 10% suilability, we eliminated ~77% of the area of Cameroon and Nigeria, area where chimpanzees do not occur [33]. The spatial extent of the mask had no influence on the relative contributions of each of the predictor variables to the models, and was used only as an extent for testing the model and displaying the results.

We identified confounding spatial variables by Pearson correlation tests that were performed using the R software package (http://www.r-project.org) using the topography, vegetation and climate data layers. We clipped each data layer to the extent of the estimated chimpanzee distribution mask. This revealed that several of these variables were highly cross-correlated (Additional file 5). Variables were considered highly correlated for r^2^ > 0.8. Only BioClim variables [63] exhibited significant cross-correlation with one another. This resulted in Bio 9, 11, 14 and 16 being excluded from the analysis. None of the topographic variables or vegetation indices exhibited significant cross-correlations and were retained for final model construction (Additional file 5).

Incorporating both environmental variables and landscape features into the GDM approach is necessary to estimate how they affect the partitioning of genetic variation and gene flow. River data layers were generated using the hydrography layer [62], to simulate the Sanaga and Mbam Rivers as complete dispersal barriers. In order to imitate a complete dispersal barrier, we coded these variables as 0 on one side, and 1 on the other side. These layers were generated for the Sanaga and Mbam Rivers using ArcMap version 10 (ESRI Corp., Redlands, CA).

We also used resistance surfaces to understand how various landscape features affect gene flow between populations by using an approach that is rooted in circuit theory and implemented in the program CIRCUITSCAPE [69]. CIRCUITSCAPE-based resistance surfaces predict dispersal routes based on predicted cost of travel. These resistance surfaces predict and quantify connectivity between pathways along multiple pathways by using cost weighted distance and suitability of the study area to determine (*i*) a cost-effective route between locations and (*ii*) a matrix of pairwise values representing the incurred cost of travel between locations [27,70]. CIRCUITSCAPE simultaneously integrates all possible pathways that connect populations [69], and has been shown to accurately predict patterns of genetic diversity among animals [27,69,71]. The resistance matrix generated for this study incorporates several variables, including habitat suitability, as determined by the ENM [33], and the size and strength of riverine barriers, based on the Strahler stream order of the major rivers in Cameron and Nigeria [62,72]. In the resistance surface used in this study, low levels of habitat suitability and larger sized rivers between sample locations generated higher levels of resistance.

### Spatial modeling

We used GDM [36] to quantify how much of the variation in the genetic data set could be explained by variation in the environmental data sets, as well as to create explicit spatial predictions of these observed patterns. Using this approach involves incorporating predicted species distributions, environmental data layers, resistance surfaces, and straight-line geographic distance as different predictors. We also used GDM to complete matrix regressions that fit nonlinear relationships between these variables. We then used the model to predict spatial patterns of genetic variation, which facilitated evaluating how environmental data may contribute to generating patterns of genetic diversity [36,73]. This resulted in a continuous spatial prediction of genetic variation across the study area. This spatial prediction was generated using metric multidimensional scaling using 5000 random sample points across the study area, with scores at neighboring pixels achieved by a *k*-nearest neighbors interpolation [36]. We ran GDM’s using the entire data set for both microsatellite and mtDNA markers, and spatial interpolation maps were generated for the entire training mask of suitable habitat for chimpanzees across Cameroon and eastern Nigeria, using the one population MAXENT model [33]. For each genetic data set, we ran various models using different groups of predictor variables, including: (1) environmental variables only, (2) environmental variables and geographic distance, and (3) environmental variables, geographic distance and riverine barriers.

We ran additional GDM’s for individual population subsets for two- and three-population models as identified by a previous study [23]. In the two-population grouping, we grouped sample locations according to their classification as originating north (*P. t. ellioti*) or south of the Sanaga River (*P. t. troglodytes*). The three-population grouping included sample locations south of the Sanaga River (*P. t. troglodytes*) and we further subdivided presence points north of the Sanaga River into two groups, *P. t. ellioti* (Rainforest) and *P. t. ellioti* (Ecotone). We only ran the GDM for the *P. t. troglodytes* group once, as this group was composed of identical sampling locations. The training masks for all GDM’s using the two- and three-population groups were obtained from the MAXENT models of corresponding groups [33].

## **Additional files**

Additional file 1
**Matrix of microsatellite pairwise differences (** ***F***_***ST***_ **).** All values in bold were determined as significant by 10,000 permutations of the data in Arlequin [57]. Additional file 2
**Matrix of mtDNA pairwise differences.** All values in bold were determined as significant by 10,000 permutations of the data in Arlequin [57]. Additional file 3
**Matrix of mtDNA Tamura and Nei differences.**
Additional file 4
**List of environmental predicting variables.**
Additional file 5
**Results from Pearson Correlation test comparing environmental variables.** Values shaded grey show all values above 0.8. Values shaded grey and bold show all values above 0.9 and had a p < 0.001.
