# Supplementary figures and images for: Environmental variation and rivers govern the structure of chimpanzee genetic diversity in a biodiversity hotspot
Source: BMC Evol Biol. 2015 Jan 21;15(1):1. doi: 10.1186/s12862-014-0274-0 (PMC4314796; doi:10.1186/s12862-014-0274-0)

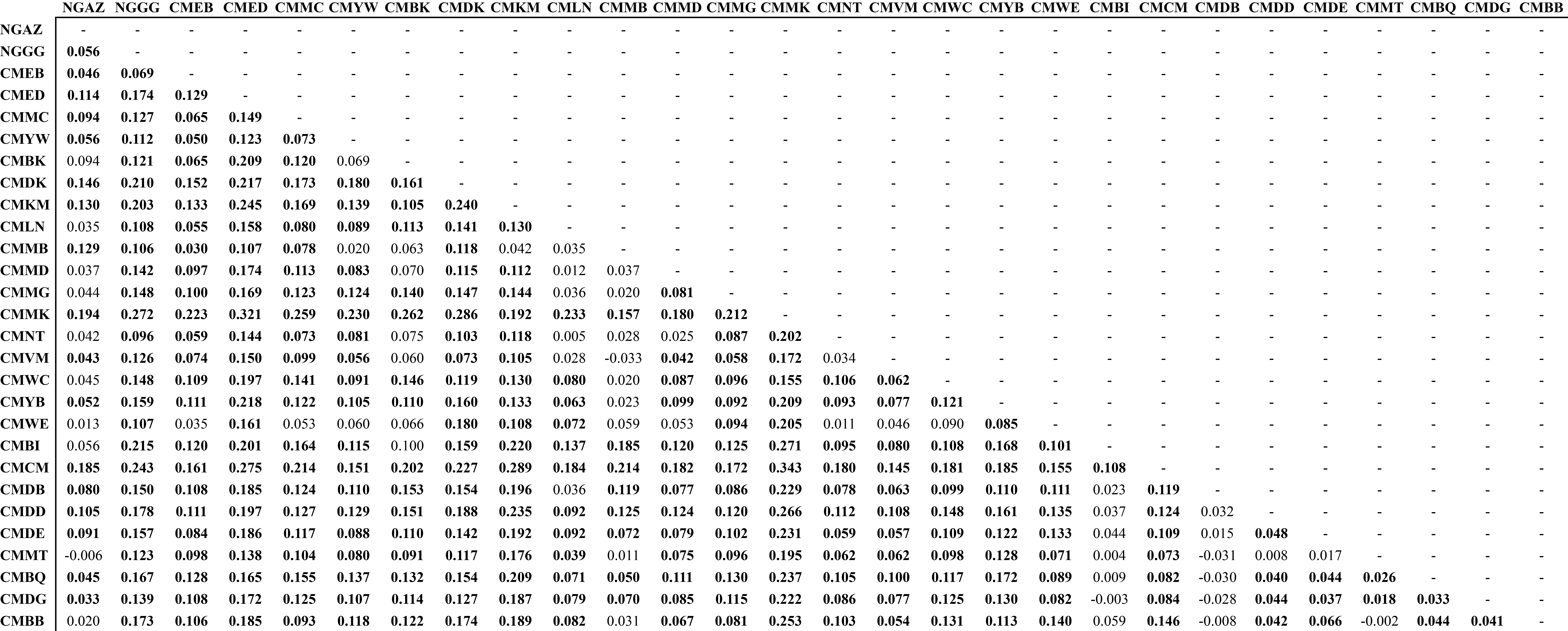

Supplement: Additional file 1 — Matrix of microsatellite pairwise differences ( F ST ). All values in bold were determined as significant by 10,000 permutations of the data in Arlequin [57]. [file 12862_2014_274_MOESM1_ESM.png]

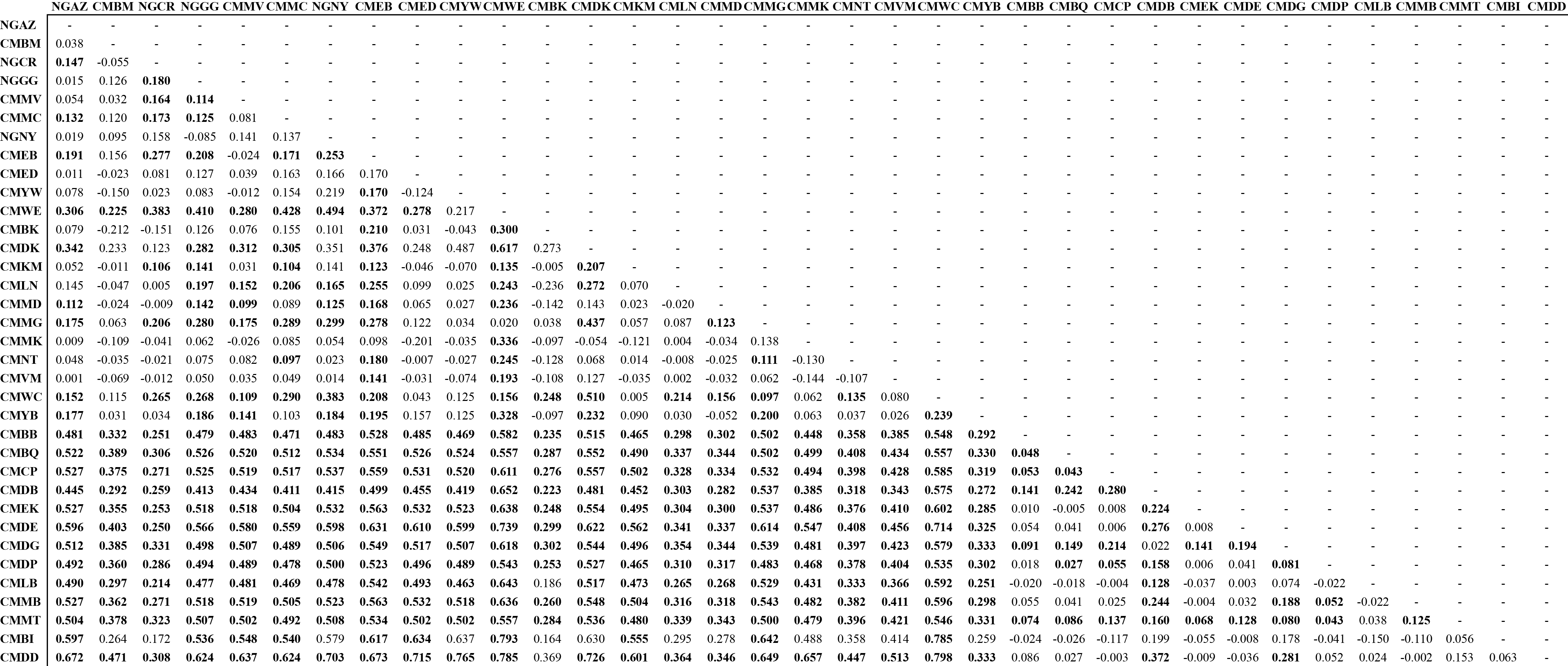

Supplement: Additional file 2 — Matrix of mtDNA pairwise differences. All values in bold were determined as significant by 10,000 permutations of the data in Arlequin [57]. [file 12862_2014_274_MOESM2_ESM.png]

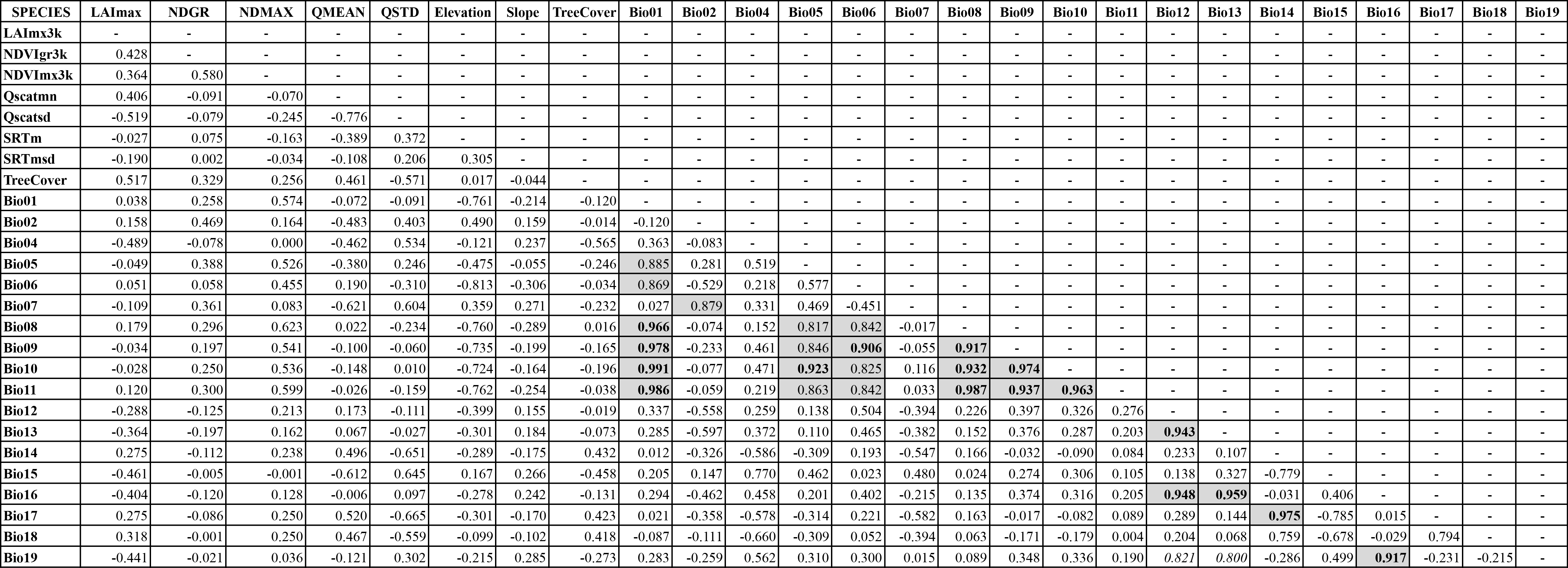

Supplement: Additional file 5 — Results from Pearson Correlation test comparing environmental variables. Values shaded grey show all values above 0.8. Values shaded grey and bold show all values above 0.9 and had a p < 0.001. [file 12862_2014_274_MOESM5_ESM.png]
