# Supplementary material for: Environmental variation and rivers govern the structure of chimpanzee genetic diversity in a biodiversity hotspot
Source: BMC Evol Biol. 2015 Jan 21;15(1):1. doi: 10.1186/s12862-014-0274-0 (PMC4314796; doi:10.1186/s12862-014-0274-0)
Supplement: Additional file 4 — List of environmental predicting variables. [file 12862_2014_274_MOESM4_ESM.docx]

| **Variable Type** | **Variable Name** | **Source** |
| --- | --- | --- |
| **Topographic Factors** | Elevation | NASA SRTM; [59] |
|  | SRTMstd – Slope (Ruggedness) |  |
|  | Slope (Degrees) | NASA SRTM; [33] |
|  | Hydrography | HydroSHEDS; [60] |
| **Climatic Factors** | Bio 1 – Annual Mean Temperature | WorldClim; [61] |
|  | Bio 2 – Mean Diurnal Range |  |
|  | Bio 4 – Temperature Seasonality |  |
|  | Bio 5 – Max. Temp. of the Warmest Month |  |
|  | Bio 6 – Min. Temp. of the Warmest Month |  |
|  | Bio 7 – Temperature Annual Range |  |
|  | Bio 8 – Mean Temp. of the Wettest Quarter |  |
|  | Bio 9 – Mean Temp. of the Driest Quarter |  |
|  | Bio 10 – Mean Temp. of the Warmest Quarter |  |
|  | Bio 11 – Mean Temp. of the Coldest Quarter |  |
|  | Bio 12 – Annual Precipitation |  |
|  | Bio 13 – Precipitation of the Wettest Month |  |
|  | Bio 14 – Precipitation of the Driest Month |  |
|  | Bio 15 – Precipitation Seasonality |  |
|  | Bio 16 – Precipitation of the Wettest Quarter |  |
|  | Bio 17 – Precipitation of the Driest Quarter |  |
|  | Bio 18 – Precipitation of the Warmest Quarter |  |
|  | Bio 19 – Precipitation of the Coldest Quarter |  |
| **Vegetation Indices** | LAImax – Leaf Area Index | MODIS; [63] |
|  | NDMAX – Max. Annual NDVI | MODIS; [27] |
|  | NDMEAN – Mean Annual NDVI |  |
|  | NDGR – Max. NDVI of Greening Season |  |
|  | NDBR – Max. NDVI of Least Green Season |  |
|  | NDGRBR – NDVI Seasonality |  |
|  | QMEAN – QSCAT Annual Mean | Quick Scatterometer; [64] |
|  | QSTD – QSCAT Standard Deviation |  |
|  | Percent Tree Cover | MODIS; [62] |
